# Beyond Screen Time: Stress, Loneliness, Emotional Competence and Problematic Internet Use in Adolescence

**DOI:** 10.3390/healthcare14080986

**Published:** 2026-04-09

**Authors:** Roberta Matković, Lucija Vejmelka

**Affiliations:** 1 Mental Health Department, Teaching Institute for Public Health of Split—Dalmatia County, 21000 Split, Croatia; roberta.matkovic@nzjz-split.hr; 2 Department of Social Work, Faculty of Law, University of Zagreb, 10000 Zagreb, Croatia

**Keywords:** problematic internet use (PIU), screen time, stress, loneliness, emotional competence

## Abstract

**Highlights:**

**What are the main findings?**

- Problematic internet use (PIU) in adolescents is explained not only by overall time online, but also by the type of online activity and adolescents’ psychological functioning (stress, loneliness, emotional competence).
- Stress and loneliness act as key risk mechanisms linking online engagement with PIU, while emotional competence functions as a protective factor, particularly in the context of social media use.

**What are the implications of the main findings?**

- Prevention should move beyond generic screen-time limits and instead target modifiable mechanisms, especially stress reduction, loneliness prevention, and strengthening self-regulation.
- School- and community-based interventions that build emotional competence and adaptive coping may reduce PIU risk and support healthier, developmentally appropriate digital engagement.

**Abstract:**

**Background:** Problematic Internet use (PIU) among adolescents has emerged as a significant public health concern, associated with the types of online activities and underlying psychological processes rather than screen time alone. **Methods:** This cross-sectional study included 750 adolescents (46.4% female) with a mean age of 15.39 years (SD = 1.76; range = 13–19) recruited from 7th and 8th grade primary school students and secondary school students in Split-Dalmatia County (Croatia). The study investigated the associations between specific online activities, psychological variables, and PIU using hierarchical regression and multiple mediation analyses. **Results:** Results revealed that time spent online remains the most strongly associated with PIU. While streaming and online shopping showed significant associations with problematic use, reading and browsing for information did not. Stress and loneliness were identified as variables associated with higher that significantly statistically mediate the relationships between online engagement and PIU, whereas emotional competence functioned as a protective factor, particularly in relation to social media use. These findings support theoretical models that conceptualize PIU as a maladaptive coping strategy for psychological distress. **Conclusions:** Based on these findings, prevention efforts should move beyond simple screen-time reduction and focus on strengthening adolescents’ emotional competence, stress management, and self-regulatory skills to promote healthier and more adaptive patterns of digital engagement.

## 1. Introduction

Digital technologies have become deeply embedded in adolescents’ everyday life positioning problematic internet use as growing public health and scientific concern that calls for systematic solutions [1]. Adolescents represent one of the most active, yet simultaneously most vulnerable, groups of internet users, with internet engagement becoming a normative part of daily life from an early age. In Europe, internet use is particularly ubiquitous among younger cohorts. Eurostat data indicate that in 2024 as many as 97% of young population aged 16–29 in the EU used the internet daily, compared with 88% in the general population [2]. In Croatia, this figure reaches almost 99% among adolescents, accompanied by pronounced generational digital inequalities. Furthermore, Croatia remains among the EU countries with the lowest household internet access rates at 88%, highest percentage of individuals who never use the internet (10%) and with internet usage in rural areas below 75% [3]. The results of the latest wave of the EU Kids Online Croatia study additionally confirmed that children from Croatia aged 8 to 10 already deeply immersed in the digital world [4]. Evidence from EU Kids Online further suggests that children are introduced to independent digital use at increasingly early ages, often without sufficient adult guidance. This report indicates that nearly 60% of children at this developmental stage have substantial opportunities to use screens and access the internet without direct adult supervision or control [4]. This suggests that Croatian adolescents often act as digital frontrunners within their families, frequently without adequate adult supervision or guidance, which may further increase their vulnerability to problematic patterns of internet use [2,5]. Recent public health monitoring further signals increasing concern regarding dysregulated online engagement among youth. The WHO Regional Office for Europe reported a rise in problematic social media use from 7% (2018) to 11% (2022) among adolescents [6]. These trends highlight the importance of distinguishing between functional and maladaptive patterns of online engagement, particularly during adolescence, a developmental period characterized by heightened sensitivity to environmental influences and increased risk-taking behaviors [5,6].

Despite extensive research interest, terminology and diagnostic boundaries remain debated. In this article, we use the concept of problematic internet use (PIU) as a broad umbrella term referring to difficulties in controlling internet-related behavior that are associated with clinically relevant distress and/or impairment in everyday functioning [7,8,9]. PIU is typically understood as heterogeneous, ranging from generalized, diffuse overuse to difficulties tied to specific online activities [8,10]. Contemporary theoretical perspectives emphasize that PIU cannot be explained solely by time spent online, but rather emerges through complex interactions between individual vulnerabilities, affective and cognitive processes, and the functional role that internet use serves in everyday life [7,10,11].

The most influential theoretical framework in this field, the Interaction of Person–Affect–Cognition–Execution (I-PACE) model, proposes that PIU emerges as a result of interactions between individual predisposing factors and affective and cognitive responses to specific online stimuli [12,13]. The I-PACE model conceptualizes PIU as a dynamic process shaped by predisposing characteristics, emotional and cognitive responses to situational triggers, and executive control processes [12,13]. Complementing this perspective, the Compensatory Internet Use Theory (CIUT) further posits that individuals engage in online activities to alleviate negative emotional states or to satisfy psychological needs that remain unmet in offline contexts [7]. Within this framework, stress and loneliness are typically conceptualized as risk-related mechanisms that may increase reliance on online activity for coping, whereas emotional competence: the ability to perceive, understand, express, and regulate emotions, may function as a protective factor [13].

Specific online activities differ in their association with PIU, where social media engagement [13,14] and online shopping [15,16] are frequently linked to self-regulatory difficulties and compulsive tendencies, whereas instrumental activities like reading and information seeking are generally considered lower-risk [17]. Stress and loneliness have been consistently associated with increased reliance on online environments as a coping strategy and with higher risk of PIU [7,18,19]. In contrast, emotional competence, encompassing emotional awareness, regulation, and expression has been identified as a protective factor supporting adaptive coping and self-regulation [13].

Furthermore, these individual processes are embedded within family contexts. Recent evidence suggests that parental interference and parenting styles are significantly associated with adolescents’ psychological distress and burnout [20,21], where supportive practices may function as protective factors Together, these frameworks highlight the importance of integrating behavioral (“what adolescents do online”) and psychological (“why they do it”) dimensions in understanding PIU. Although previous studies have examined psychological correlates of PIU, relatively few have simultaneously integrated differentiated online activities, exposure patterns, and multiple psychological mechanisms within a single model, particularly in under-researched regional contexts. To date, such integrative approaches remain limited in Southeast European settings, including Croatia. The present study addresses this gap by examining PIU among adolescents through a lens that combines (a) internet exposure, (b) specific online activities, and (c) psychological factors (stress, loneliness, and emotional competence). Grounded in the I-PACE model and CIUT, this study was designed to examine the expected psychological and behavioral pathways linking adolescents’ online activities to problematic internet use.

### Aim and Hypotheses

The study aimed to examine the associations and potential psychological mechanisms linking adolescents’ internet exposure, specific online activities, and psychological factors with problematic Internet use (PIU). Based on the theoretical framework, the following hypotheses were formulated:

**H1.**  *Greater overall time spent online, as well as more frequent engagement in social media use, online shopping, and streaming activities, would be positively associated with PIU.*

**H2.**  *Higher levels of perceived stress and loneliness will be positively associated with PIU and may partially explain the relationship between online engagement and PIU.*

**H3.**  *Emotional competence will be negatively associated with PIU and may buffer the adverse effects of stress and loneliness on problematic Internet engagement [7,12,13].*

## 2. Materials and Methods

### 2.1. Ethical Considerations

The study was conducted with the approval of the Ministry of Science, Education and Youth (Croatia), as well as the Ethics Committee of the Public Health Institute of Split-Dalmatia County (Split). During the preparation and implementation of the study, the Code of Ethics in Research with Children was adhered to. Parents were informed about the implementation of the study and provided their written informed consent for their children’s participation [22]. Students were also informed about the purpose of the study prior to completing the questionnaire. In addition, students retained the right to refuse participation at any time, either before or during the research process.

### 2.2. Study Design and Participants

The study included students attending the 7th and 8th grades of primary schools and the 1st to 4th grades of secondary schools in the largest county in Croatia. A stratified cluster sampling design was applied (islands vs. mainland, municipality vs. city, and for secondary schools, educational type (grammar schools, four-year vocational programs, and three-year vocational programs), with schools within clusters and classes within schools selected at random. A total of 23 schools were initially selected; however, one school withdrew prior to data collection and was not replaced, resulting in a final sample of 22 participating schools. School principals provided institutional consent, and in collaboration with school staff, one educational track per school was selected, with all classes within that track included in the study.

A cross-sectional study design with a single measurement point was employed. Data were collected using a survey questionnaire created via an online form, which students completed during regular class time in the presence of a teacher or school counselor. Completion required one school class (maximum of 45 min). The questionnaire was a structured, self-report instrument consisting of standardized scales and study-specific items. Data collection was conducted between April and June 2025.

A total of 1296 students were eligible to participate in the study. Of these, 750 were completed (response rate = 57.9%). Non-participation was due to parental or student refusal (n = 341; 26.3%) and student absence on the day of data collection (n = 205; 15.8%). No additional exclusion criteria were applied beyond non-consent and absence.

This paper presents a subset of these findings, with a particular focus on PIU and indicators of stress, loneliness, and emotional competencies from the broader study aimed at exploration of behavioral problems among adolescents, such as PIU and problematic gambling, as well as indicators of mental health, sleep quality, and emotional competencies.

### 2.3. Measures

The measures included sociodemographic variables (age, gender, grade, and education type), indicators of internet use (time and types of activities), psychological variables (stress, loneliness, and emotional competence), and problematic internet use. All instruments were administered in Croatian. All standardized instruments used in this study had previously been translated and validated in the Croatian context; therefore, no additional validation procedures were required.

Time spent on the internet was assessed using two questions developed specifically for the purposes of this study, referring to average daily internet use during weekdays and weekends. The items were adapted from established international surveys on adolescent online behavior, including the European School Survey Project on Alcohol and Other Drugs (ESPAD) and the EU Kids Online study conducted in Croatia, with minor modifications to the response categories to better reflect current patterns of internet use [23,24]. These items were developed based on previously used instruments and were reviewed for clarity and suitability for adolescent participants prior to administration. Participants estimated how much time per day they spent on the internet during the week (Monday to Friday) and during the weekend (Saturday and Sunday), and were instructed to include the use of all devices (not only mobile phones) and all types of online content (e.g., messaging, social networking, gaming, listening to music, etc.). Responses were given on a 10-point scale ranging from 0 = I do not use the internet to 9 = more than 8 h. Higher results indicate a longer average daily time spent on the internet. The two items were analyzed as separate indicators of internet use (weekday and weekend), rather than being combined into a composite score or averaged. This approach was chosen to preserve potential differences in usage patterns between weekdays and weekends, rather than aggregating them into a single index. Additionally, the distribution of responses (Table 1) indicated that midpoint categories were meaningfully used by participants. For completeness, the two items demonstrated good internal consistency (Cronbach’s α = 0.834) in the present study.

Use of specific content. A questionnaire was developed specifically for the purposes of this study. Participants reported how frequently they used social media, the internet for reading, browsing, and searching for information, streaming/downloading music, videos, and films, and searching for, selling, or purchasing products such as goods, clothing and footwear, etc., during the past 30 days. Responses were given on a 5-point scale ranging from 0 = never to 4 = almost always (at least 6 days per week). The items were reviewed for clarity and age-appropriateness prior to data collection. Each activity was assessed using a single item and analyzed as an independent variable, reflecting distinct types of online activities rather than combined into a composite score. Exploratory factor analysis was conducted for descriptive purposes only (KMO = 0.78; Bartlett’s test *p* < 0.001) and indicated a single-factor solution explaining 63.14% of the variance. Although a total score was not used in the present analyses, the internal consistency of the four items was examined for descriptive purposes only and indicated acceptable reliability (Cronbach’s α = 0.803).

Stress. Stress was assessed using the Stress subscale of the Depression, Anxiety, and Stress Scale (DASS-21) [25]. The scale was translated and culturally adapted into and has been previously applied in the Croatian context [26]. The subscale consists of seven items (e.g., “I felt that I was getting agitated,” “I found it difficult to relax”), and participants indicated the extent to which each statement applied to them over the past week on a 4-point scale (0 = did not apply to me at all, 3 = applied to me most of the time). The stress subscale score was calculated by summing responses to all seven items and multiplying the total by two, with higher scores indicating higher levels of stress. In this study, the stress subscale demonstrated high internal consistency, with Cronbach’s alpha (α) of 0.918.

Loneliness was measured using a shortened version of the UCLA Loneliness Scale [27]. The scale was translated and culturally adapted into and has been previously applied in the Croatian context [28,29,30]. The scale consists of seven items (e.g., “I do not share my opinions and ideas with others,” “No one really knows me”), which participants rated on a 5-point scale (1 = does not apply to me at all, 5 = applies to me completely). The total score was calculated as the sum of responses to all seven items, with higher scores indicating greater loneliness. The scale demonstrated strong internal reliability, with Cronbach’s alpha (α) of 0.906.

The Emotional Competence Questionnaire—UEK-15 is a shortened version of the original UEK-45 and consists of 15 items (e.g., “I learn from unpleasant experiences how one should not behave in the future,” “With friends, I can distinguish when they are sad and when they are disappointed”) [31]. The instrument assesses multiple components of emotional competence, including the perception, understanding, recognition, expression, regulation and management of emotions. It has been previously validated and applied in adolescent populations (e.g., [32]). Participants rated the extent to which each item applied to them on a 5-point scale ranging from 1 = not at all to 5 = completely. The scale was designed as a single dimensional measure, with the total score calculated as the sum of responses to all 15 items where higher scores indicate higher emotional competence. The scale showed strong internal reliability, with Cronbach’s alpha (α) of 0.961.

The Internet Addiction Test consists of 20 items [9]. The instrument has been translated and validated in the Croatian context [33] and has been widely applied in studies involving adolescents, supporting its suitability for this population [34,35,36,37]. Participants assessed how much the described statements applied to them over the past month (e.g., “How often do you use the Internet longer than you intended?”, “How often do you neglect household chores in order to spend more time online?”). Responses were given on a 6-point scale ranging from 0 (never/not applicable) to 5 (always). The total score is calculated as the sum of responses to all 20 items and can range from 0 to 100, with higher total scores indicating a higher level of Internet addiction. The scale showed strong internal reliability, with Cronbach’s alpha (α) of 0.978. Although the instrument is traditionally referred to as the *Internet Addiction Test*, it was used in the present study addressed as a general measure of problematic Internet use (PIU), given the conceptual overlap and ongoing lack of consensus regarding the classification of Internet addiction, as well as the absence of its formal recognition in major diagnostic systems [38,39]. The scale captures a broad range of maladaptive Internet-related behaviors and associated functional impairments, rather than assessing *Internet addiction* as a formally established diagnostic category, which is not currently recognized in clinical classification systems.

In addition to scales specifically developed for the purposes of this study, all other measurement instruments that are not openly and freely available were used with prior permission obtained from the original authors.

In Table 1, descriptive statistics are presented for patterns of Internet use, and psychological measures included in the study.

### 2.4. Data Analysis

Normality of the dependent variable, as assessed by the Shapiro–Wilk test, indicated a statistically significant deviation from a normal distribution (*p* < 0.001), which is expected given the large sample size (n = 750). The absolute values of skewness (1.13) and kurtosis (0.81) indicate only minor deviations from normality. In accordance with methodological recommendations, the distribution does not exhibit extreme non-normality and can therefore be treated as approximately normal [40,41].

To examine the contribution of predictors to problematic internet use, a hierarchical regression analysis was conducted. Prior to the analysis, key assumptions of regression were tested, including the level of measurement of variables, adequacy of sample size, normality of residuals, linearity of relationships, and multicollinearity [42]. The variables included in the analysis were measured at an ordinal level but were treated as approximately continuous. The sample size (n = 750) was considered sufficient given the number of predictors included. Normality of residuals was assessed using a P–P plot, which indicated that the distribution followed the expected normal line. Multicollinearity was not detected, as all VIF values were below 5. Predictors were not standardized prior to the regression analysis. Standardized regression coefficients (β) were used for interpretation.

All mediation analyses were conducted using PROCESS macro Model 6, which allows for the simultaneous estimation of direct effects, indirect effects, and serial in-direct effects [42]. Statistical significance of indirect effects was evaluated using a bootstrap procedure with 5000 resamples, and 95% bias-corrected confidence intervals were calculated. Indirect effects were considered statistically significant when the confidence interval did not include zero.

Covariates were not initially included in the mediation models. The decision to include sociodemographic variables (age and gender) as control variables was planned to be based on the results of the hierarchical regression analysis. If age and gender were found to significantly contribute to the explanation of variance in problematic internet use, they would be included as covariates in the mediation models. As these variables were not the primary focus of the study, they were planned to be excluded in the case of non-significant effects in order to maintain model simplicity.

## 3. Results

A total of n = 750 students participated in the study, of whom 46.4% (n = 348) were female and 53.6% (n = 402) were male, with a mean age of M = 15.39 years (SD = 1.76, min = 13, max = 19). Primary school students (7th and 8th grades) comprised 36.7% of the sample (n = 275), students enrolled in three-year secondary vocational programs 8.3% (n = 62), those in four-year secondary vocational programs 32.8% (n = 246), and grammar school students 22.3% (n = 167). Participants attended schools located in Split (34.4%, n = 258), the largest city and administrative center of Split-Dalmatia County, as well as in smaller towns (45.9%, n = 344), municipalities (8.4%, n = 63), and island communities (11.3%, n = 85).

Pearson correlation analyses (Table 2) indicated that different types of online activities engaged in during the past 30 days were moderately to strongly positively interrelated (r = 0.41–0.60), indicating moderate to strong co-occurrence of online activities. The strongest associations were observed between reading/browsing and streaming (r = 0.60), reading/browsing and online shopping (r = 0.56), and social media use and reading/browsing (r = 0.54). Time spent online during weekdays and weekends showed a very strong positive correlation (r = 0.72), indicating stable patterns of Internet use across the week. Both weekday and weekend Internet use were moderately associated with all specific online activities, including social media use (r = 0.35–0.43), reading/browsing (r = 0.28–0.32), streaming (r = 0.27–0.31), and online shopping (r = 0.34–0.37).

All examined online activities were significantly positively associated with PIU, as measured by the Internet Addiction Test (IAT), with correlation coefficients ranging from r = 0.32 to r = 0.39. Among psychological variables, stress showed consistent positive associations with all forms of online activity (r = 0.12–0.21) and exhibited the strongest correlation with PIU (r = 0.41). Loneliness was positively associated with PIU (r = 0.35) and showed a small but significant association with streaming behavior (r = 0.11).

Emotional competence was positively associated with the frequency of online activities, particularly social media use (r = 0.31), but negatively associated with stress (r = −0.14), loneliness (r = −0.11), and PIU (r = −0.08). Stress and loneliness were positively associated with time spent online during both weekdays (r = 0.24 and r = 0.14, respectively) and weekends (r = 0.32 and r = 0.17, respectively). Emotional competence was not significantly associated with the amount of time spent online.

### 3.1. Explaining Problematic Internet Use: A Hierarchical Regression Approach

To examine the extent to which sociodemographic variables, time spent online, patterns of Internet use, and psychological variables explain PIU, a hierarchical regression analysis was conducted (Table 3).

In the first step, sociodemographic variables (gender and age) were entered into the model. This initial model explained 1.9% of the variance in PIU, with gender emerging as a significant predictor, whereas age did not contribute significantly. In the second step, time spent online during weekdays and weekends was added to the model, increasing explained variance to 26.1%. Both weekday and weekend Internet use emerged as significant positive predictors of PIU, indicating that greater overall Internet exposure is strongly associated with higher levels of problematic use. The third step included specific types of online activities during the past 30 days (social media use, reading/browsing and information seeking, streaming/downloading music, videos, or movies, and online shopping or selling). This step increased explained variance by 6.0%, increasing the total explained variance to 32.1%. Within this model, streaming/downloading music, videos, or movies and online shopping or selling emerged as significant predictors, whereas social media use and reading/browsing for information did not contribute significantly. In the final step, psychological variables (stress, loneliness, and emotional competence) were entered into the model, increasing total explained variance to 41.5% of PIU (F (11, 738) = 47.67, *p* < 0.001). In this final model, stress and loneliness emerged as significant positive predictors, while emotional competence emerged as a significant negative predictor of PIU. After inclusion of psychological variables, streaming/downloading music, videos, or movies was no longer a significant predictor, while social media use became significant. Time spent online remained a significant predictor across all stages of the model.

### 3.2. Psychological Mechanisms Linking Online Activities to Problematic Internet Use: Mediation Analyses

Because online shopping or selling showed a consistent association with PIU, whereas streaming ceased to be significant after the inclusion of psychological variables and social media use became significant only after psychological variables were entered, three multiple mediation analyses were conducted to examine the mediating roles of perceived stress, loneliness, and emotional competence in the relationships between specific online activities and PIU. As age and gender did not show a statistically significant contribution in the hierarchical regression analysis, they were not included as covariates in the mediation analyses.

#### 3.2.1. Mediation Model 1: Streaming/Downloading Music, Videos, or Movies

**Figure 1:**
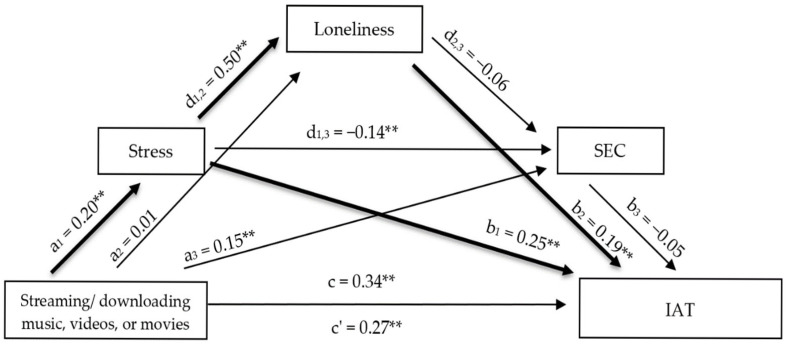
Serial mediation model examining the indirect effects of streaming/downloading music, videos, or movies on PIU through perceived stress, loneliness, and emotional competence. Note. Standardized regression coefficients (β) are shown. ** *p* < 0.01; IAT = Problematic Internet Use.

The mediation model examining the relationship between streaming/downloading music, videos, or movies and PIU explained 26.59% of the variance. The analysis revealed a statistically significant total effect of streaming behavior on PIU (Table S1). After including perceived stress, loneliness, and emotional competence in the model, the direct effect remained statistically significant, indicating partial mediation [43,44]. Streaming/downloading music, videos, or movies was significantly and positively associated with perceived stress. Perceived stress, in turn, showed a strong positive association with loneliness and a significant negative association with emotional competence. Both perceived stress and loneliness emerged as significant positive predictors of PIU, whereas emotional competence did not demonstrate a statistically significant direct effect. In addition, the association between loneliness and emotional competence was not statistically significant. The total indirect effect was statistically significant. Among the specific indirect pathways, a simple mediation through perceived stress and a serial mediation through perceived stress and loneliness were statistically significant. Indirect pathways involving emotional competence were not statistically significant (Figure 1).

#### 3.2.2. Mediation Model 2: Social Media Use

The model examining social media use accounted for 28.69% of the variance in PIU. The total and direct effect were statistically significant, indicating inconsistent mediation (Table S2) [45,46]. Social media use was significantly and positively associated with perceived stress and simultaneously positively associated with emotional competence, whereas its association with loneliness was not statistically significant. Perceived stress was strongly positively associated with loneliness. Furthermore, perceived stress, loneliness, and emotional competence all emerged as significant predictors of PIU, with stress and loneliness acting as risk factors and emotional competence acting as a protective factor.

**Figure 2:**
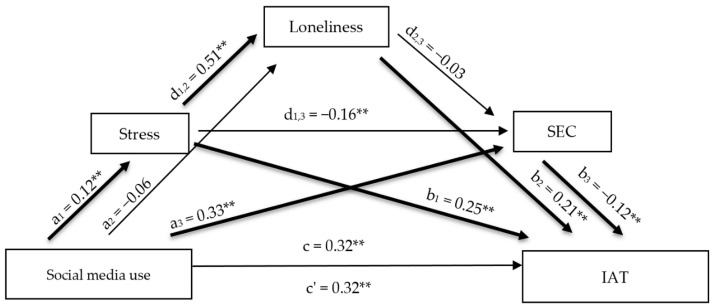
Serial mediation model examining the indirect effects of social media use on PIU through perceived stress, loneliness, and emotional competence. Note. Standardized regression coefficients (β) are shown. ** *p* < 0.01; IAT = Problematic Internet Use.

The analysis of indirect effects revealed the presence of significant specific indirect pathways, including a simple mediation through perceived stress, a simple mediation through emotional competence, and a serial mediation through perceived stress and loneliness. However, the total indirect effect was not statistically significant, due to the opposing directions of the individual indirect pathways (Figure 2).

#### 3.2.3. Mediation Model 3: Online Shopping or Selling

The third mediation model examining online shopping or selling explained 30.60% of the variance in PIU. The total effect was statistically significant, and the direct effect remained significant after including the mediators, indicating partial mediation (Table S3) [43,44].

Online shopping or selling was significantly and positively associated with perceived stress, while its association with loneliness was not statistically significant. It also showed a significant positive association with emotional competence. Perceived stress exhibited a strong positive association with loneliness and a significant negative association with emotional competence. Both perceived stress and loneliness emerged as significant positive predictors of PIU, whereas emotional competence did not show a statistically significant direct effect on PIU.

**Figure 3:**
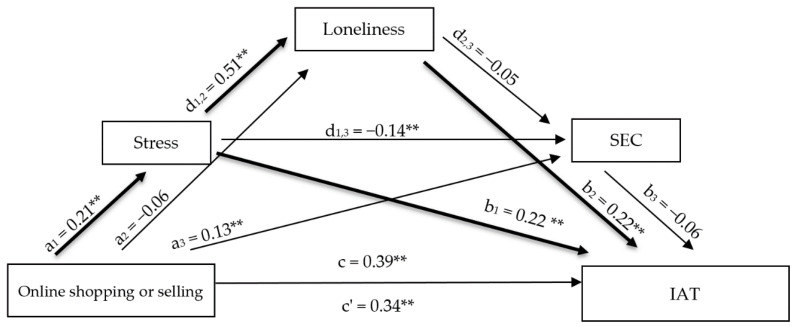
Serial mediation model examining the indirect effects of online shopping or selling on PIU through perceived stress, loneliness, and emotional competence. Note. Standardized regression coefficients (β) are shown. ** *p* < 0.01; IAT = Problematic Internet Use.

The analysis of indirect effects demonstrated that the total indirect effect was statistically significant. Specifically, a simple mediation through perceived stress and a serial mediation through perceived stress and loneliness were statistically significant, whereas indirect pathways involving emotional competence were not significant (Figure 3).

## 4. Discussion

The present findings extend previous research by integrating activity-specific behaviors and multiple psychological mechanisms within a single explanatory framework. Consistent with contemporary theoretical approaches, particularly the Interaction of Person–Affect–Cognition–Execution (I-PACE) model and the Compensatory Internet Use Theory that problematic Internet use (PIU) among adolescents cannot be reduced to time spent online only [7,13]. Rather, PIU appears to be associated with the quality, purpose, and psychological context of Internet use, reinforcing the view that maladaptive online behavior is closely intertwined with processes of emotional regulation and coping.

Adolescent Internet use in this study was characterized by high engagement across multiple online activities and relatively stable patterns of use across weekdays and weekends, suggesting that online engagement represents a routine component of everyday life. Importantly, PIU was more strongly associated with overall time spent online and psychological difficulties, particularly stress and loneliness. These findings are consistent with prior research indicating that psychological distress and self-regulatory difficulties are closely linked to problematic patterns of Internet use [18,19,20]. Adolescents with higher emotional competence did not necessarily spend less time online, but they were less likely to exhibit problematic use patterns This finding aligns with emotion regulation perspectives, which indicate that adaptive regulatory capacities reduce reliance on avoidance-based coping strategies, including excessive engagement in digital environments [13,47,48]. Accordingly, emotional competence appears to be more closely related to the regulation and quality of Internet use rather than to its quantity. Importantly, given the cross-sectional nature of the data, these findings should be interpreted as associations rather than causal relationships. However, the findings also demonstrate that specific online activities and psychological variables make meaningful and independent contributions, underscoring the importance of considering both behavioral and psychological dimensions when examining adolescents’ Internet-related difficulties.

Findings from the regression analyses further highlight the importance of both behavioral and psychological factors. Total time spent online emerged as the most stable and robust predictor of PIU across all models, suggesting that increased exposure is consistently associated with higher levels of problematic use. At the same time, psychological variables remained significant after controlling for time, indicating that distress-related processes may increase vulnerability within the context of high exposure.

With regard to specific online activities, differential patterns emerged. Social media use was associated with PIU only after psychological variables were included in the model, whereas streaming content, whereas streaming content lost its significance in the final model. These findings suggest that the association between these activities and PIU is contingent upon adolescents’ psychological functioning, particularly levels of stress, loneliness, and emotional competence. Such results align with previous evidence indicating that online environments can serve both adaptive and maladaptive functions, depending on users’ emotional needs and regulatory capacities [7,49].

In contrast, reading and browsing for information were not significantly associated with PIU at any step of the analysis, supporting their conceptualization as instrumental, goal directed Internet use with a lower addictive potential [17]. Conversely, online shopping remained significantly associated with PIU even after controlling for psychological variables, suggesting a more activity-specific risk profile. This pattern is consistent with prior research linking online shopping to impulsivity, reward sensitivity, and compulsive behavioral tendencies [15,16,50,51]. Across all regression models, stress and loneliness emerged as significant risk factors, while emotional competence consistently functioned as a protective factor. This constellation of findings is fully consistent with I-PACE assumptions that PIU represents a maladaptive form of affect regulation in individuals with heightened psychological vulnerability [13].

Finally, the findings should be interpreted within broader developmental and contextual frameworks. Gender-specific patterns of online engagement and coping should be considered, as previous studies indicate that the relationship between Internet use and psychological distress may vary across gender and types of online activities [52,53]. In addition, parental mediation and family dynamics are relevant contextual factors, as different parenting practices have been associated with adolescents’ emotional regulation and psychological well-being [20,21]. In line with the present findings, it is plausible that family environments that support emotional development and autonomy contribute to more adaptive patterns of Internet use, whereas more controlling or intrusive parenting may be linked to increased psychological vulnerability and, consequently, higher risk of problematic use. Furthermore, the Croatian context characterized by a pronounced digital divide between younger and older generations [3] it may further shape adolescents’ autonomy in digital environments, often with limited parental guidance.

### Limitations and Future Directions

Several limitations of the present study should be acknowledged when interpreting the findings. The cross-sectional design precludes conclusions about causality and the temporal ordering of variables. Although the mediation analyses were theoretically grounded and statistically robust, the observed indirect effects should be interpreted as indicative of potential psychological pathways rather than definitive causal mechanisms. Longitudinal and experimental research is needed to clarify whether psychological distress precedes problematic internet use (PIU), results from it, or develops through reciprocal processes over time. The reliance on self-report measures introduces the possibility of common method bias and reporting inaccuracies, including social desirability and recall bias. Adolescents’ estimates of time spent online and engagement in specific activities may not fully reflect actual usage patterns. The future studies would benefit from incorporating objective indicators (e.g., digital trace or screen-time data) and multi-informant approaches to strengthen measurement validity. Although the sample was relatively large and diverse in terms of school type and age range, it was drawn from a single regional context. Cultural, educational, and family-related factors may shape both Internet use patterns and psychological vulnerability. Consequently, the generalizability of the findings to adolescents in other sociocultural settings may be limited. Replication in different national and cultural contexts is therefore warranted.

Data collection was conducted in classroom settings where teachers or school counselors were present to ensure appropriate conditions. Although they did not monitor individual responses, their presence may have influenced participants’ willingness to respond fully honestly and could have contributed to social desirability or perceived evaluation pressure.

While the study focused on key psychological variables: stress, loneliness, and emotional competence, other relevant factors were not included. Variables such as impulsivity, sensation seeking, depressive symptoms, parental monitoring, and peer norms may further elucidate individual differences in vulnerability to PIU and should be integrated into future models

Finally, emotional competence was examined as a general construct, and the study did not differentiate between specific components (e.g., emotional awareness, regulation strategies, empathy) or between different types of social media platforms and content. Given that these factors may vary in their psychological demands and associated risks, future research should adopt more fine-grained approaches. Despite these limitations, the present study provides a solid empirical foundation for understanding PIU as a psychologically embedded phenomenon and offers clear directions for future research.

## 5. Conclusions

The present study contributes to the growing body of literature on adolescent PIU by demonstrating that PIU is not merely a function of time spent online, but rather a complex outcome shaped by patterns of online activity and underlying psychological processes. Consistent with the I-PACE model and the Compensatory Internet Use Theory, the findings indicate that adolescents may engage in intensive online behaviors as a means of regulating stress and coping with feelings of loneliness, with problematic use emerging when such strategies become maladaptive.

Across all analyses, perceived stress and loneliness emerged as the most consistent psychological risk factors mediating the associations between specific online activities and PIU. Emotional competence, in contrast, functioned as a context-dependent protective factor, particularly in relation to social media use, highlighting the importance of considering both risk-enhancing and protective mechanisms within the same behavioral context. Notably, different online activities were associated with PIU through partially distinct psychological pathways, underscoring the need to move beyond one-size-fits-all explanations of PIU.

From an applied perspective, the findings suggest that prevention and intervention efforts should not focus solely on limiting screen time, but rather on strengthening adolescents’ emotional competence, stress management skills, and self-regulatory capacities. Interventions that help young people recognize the emotional motives underlying their online behavior and develop adaptive coping strategies may be particularly effective in reducing the risk of PIU.

## Figures and Tables

**Table 1:** Descriptive statistics for sociodemographic variables, Internet use variables, and psychological measures (n = 750).

	M	SD	Med	Mod	Min	Max
Age (years)	15.39	1.76	15	13	13	19
Time spent online on weekdays (hours per day)	5.06	2.60	5	9	0	9
Time spent online on weekends (hours per day)	5.09	2.56	5	4	0	9
Social media use	2.79	1.51	4	4	0	4
Reading/browsing and information seeking	1.88	1.47	2	0	0	4
Streaming/downloading music, videos, or movies	1.60	1.51	1	0	0	4
Online shopping or selling	1.48	1.46	1	0	0	4
Stress	11.61	11.05	10	0	0	42
Loneliness	12.93	6.57	11	7	7	35
Emotional competence	51.15	15.12	52	45	15	75
Problematic Internet Use	26.66	25.25	20	0	0	100

Note. M = mean; SD = standard deviation; Med = median; Mod = mode; Min = minimum; Max = maximum.

**Table 2:** Pearson correlations among sociodemographic variables, Internet use variables, and psychological measures (n = 750).

		1	2	3	4	5	6	7	8	9	10	11	12
1	Gender	1											
2	Age (years)	−0.093 *	1										
3	Time spent online on weekdays	0.204 **	0.142 **	1									
4	Time spent online on weekends	0.240 **	0.104 **	0.716 **	1								
5	Social media use	0.250 **	−0.052	0.349 **	00.425 **	1							
6	Reading/browsing and information seeking	0.169 **	0.064	0.277 **	0.317 **	0.539 **	1						
7	Streaming/downloading music, videos, or movies	0.047	0.070	0.271 **	0.313 **	0.412 **	0.602 **	1					
8	Online shopping or selling	0.177 **	0.126 **	0.365 **	0.344 **	0.412 **	0.562 **	0.510 **	1				
9	Stress	0.207 **	0.008	0.240 **	0.315 **	0.121 **	0.185 **	0.201 **	0.209 **	1			
10	Loneliness	−0.002	0.053	0.136 **	0.168 **	0.006	0.062	0.114 **	0.048	0.503 **	1		
11	Emotional competence	0.009	0.040	−0.020	−0.011	0.311 **	0.214 **	0.115 **	0.094 **	−0.137 **	−0.111 **	1	
12	PIU	0.132 **	0.029	0.449 **	0.490 **	0.316 **	0.323 **	0.339 **	0.393 **	0.409 **	0.352 **	−0.078 *	1

Note. Pearson’s r coefficients are reported. * *p* < 0.05; ** *p* < 0.01. Gender was coded 1 = male, 2 = female. Time spent online on weekdays and weekends is expressed in hours per day, whereas online activity variables refer to frequency of engagement during the past 30 days.

**Table 3:** Hierarchical regression analysis predicting PIU.

Predictors	1st Step		2nd Step		3rd Step		4th Step	
	β	t	β	t	β	t	β	t
Gender	0.136	3.74 **	0.004	0.12	−0.016	−0.51	−0.036	−1.20
Age	0.042	1.14	−0.036	−1.12	−0.054	−1.74	−0.047	−1.61
Time spent online on weekdays			0.206	4.53 **	0.153	3.45 **	0.139	3.36 **
Time spent online on weekends			0.345	7.57 **	0.283	6.22 **	0.200	4.65 **
Social media use					0.013	0.32	0.084	2.22 *
Reading/browsing and information seeking					0.032	0.73	0.031	0.77
Streaming/downloading music, videos, or movies					0.099	2.48 *	0.060	1.61
Online shopping or selling					0.176	4.47 **	0.172	4.66 **
Stress							0.142	4.05 **
Loneliness							0.204	6.17 **
Emotional competence							−0.085	−2.78 **
Total Model								
R^2^	0.019		0.261		0.321		0.415	
Adjusted R^2^	0.017		0.257		0.313		0.407	
ΔR^2^	0.019 **		0.242 **		0.060 **		0.095 **	

Note. * *p* < 0.05; ** *p* < 0.01; β—standardized regression coefficient; R^2^—coefficient of multiple determination; Adjusted R^2^—adjusted coefficient of multiple determination; ΔR^2^—change in coefficient of multiple determination.

## Data Availability

Data are available upon request.

## Supplementary Materials

The following supporting information can be downloaded at: https://www.mdpi.com/article/10.3390/healthcare14080986/s1, Table S1. Total, direct and indirect effects of streaming/ downloading music, videos, or movies on problematic internet use via stress, loneliness, and emotional competence. Table S2. Total, direct and indirect effects of social media use on problematic internet use via stress, loneliness, and emotional competence. Table S3. Total, direct and indirect effects of online shopping or selling on problematic internet use via stress, loneliness, and emotional competence.
