# Supplementary material for: Beyond Screen Time: Stress, Loneliness, Emotional Competence and Problematic Internet Use in Adolescence
Source: Healthcare (Basel). 2026 Apr 9;14(8):986. doi: 10.3390/healthcare14080986 (PMC13115697; doi:10.3390/healthcare14080986)
Supplement: Supplementary file 1 [file healthcare-14-00986-s001.zip › healthcare-4158872-supplementary.pdf]

Supplementary

Table S1. Total, direct and indirect effects of streaming/ downloading music, videos, or movies on problematic internet use via stress, loneliness, and emotional competence

			B	SE	LLCI	ULCI	β
	a_1	Streaming/ downloading music, videos, or movies – Stress	1.47	0.26	0.96	1.98	0.20**
	a_2	Streaming/ downloading music, videos, or movies – Loneliness	0.06	0.14	-0.22	0.33	0.01
	a_3	Streaming/ downloading music, videos, or movies –Emotional competence	1.49	0.37	0.77	2.20	0.15**
	$d_{1,2}$	Stress – Loneliness	0.30	0.02	0.26	0.34	0.50**
	$d_{1,3}$	Stress – Emotional competence	-0.19	0.06	-0.30	-0.07	-0.14**
	$d_{2,3}$	Loneliness - Emotional competence	-0.14	0.10	-0.32	0.05	-0.06
	b_1	Stress – IAT	0.57	0.08	0.41	0.74	0.25**
	b_2	Loneliness - IAT	0.73	0.14	0.45	1.00	0.19**
	b_3	Emotional competence - IAT	-0.09	0.05	-0.20	0.01	-0.05
Total effect		c	5.65	0.57	4.52	6.77	0.34**
Direct efekt		c'	4.55	0.54	3.49	5.61	0.27**
Ind1	(a_1b_1)	Streaming/ downloading music, videos, or movies – Stress –IAT	0.84	0.25	0.39	1.39	0.05*
Ind2	(a_2b_2)	Streaming/ downloading music, videos, or movies – Loneliness - IAT	0.04	0.11	−0.16	0.29	0.00
Ind3	(a_3b_3)	Streaming/ downloading music, videos, or movies – Emotional competence - IAT	−0.13	0.09	−0.29	0.05	−0.01
Ind4	($a_1d_{1,2}b_2$)	Streaming/ downloading music, videos, or movies – Stress – Loneliness - IAT	0.32	0.11	0.14	0.55	0.02*
Ind5	($a_1d_{1,3}b_3$)	Streaming/ downloading music, videos, or movies – Stress – Emotional competence - IAT	0.02	0.02	−0.01	0.07	0.00
Ind6	($a_2d_{2,3}b_3$)	Streaming/ downloading music, videos, or movies – Loneliness - Emotional competence - IAT	0.00	0.00	−0.01	0.01	0.00
Ind7	($a_1d_{1,2}d_{2,3}b_3$)	Streaming/ downloading music, videos, or movies – Stress – Loneliness - Emotional competence - IAT	0.01	0.01	0.00	0.02	0.00

Model	Stress	Loneliness	Emotional competence	IAT
R^2	0.04	0.25	0.04	0.27
F	31.56**	126.29**	11.01**	67.46**
df	(1, 748)	(2, 747)	(3, 746)	(4, 745)

B = unstandardized regression coefficient SE = standard error; t = t value; 95% CILB = lower bound of the 95% confidence interval; 95% CIUB = upper bound of the 95% confidence interval; β = standardized regression coefficient; significance level; *p < .05, **p < .01; R^2 = coefficient of determination; F = F value; df = degrees of freedom of the model and the residual ($df_{1,2}$); IAT= Internet Addiction Test; Total Indirect effect: B = 1.10. SE = 0.34, 95% CI [0.45; 1.83], β = 0.07

Table S2. Total, direct and indirect effects of social media use on problematic internet use via stress, loneliness, and emotional competence

			B	SE	LLCI	ULCI	β
	a_1	Social media use – Stress	0.89	0.27	0.36	1.41	0.12**
	a_2	Social media use – Loneliness	-0.24	0.14	-0.52	0.03	-0.06
	a_3	Social media use –Emotional competence	3.31	0.35	2.63	3.99	0,33**
	$d_{1,2}$	Stress – Loneliness	0.30	0.02	0.27	0.34	0.51**
	$d_{1,3}$	Stress – Emotional competence	-0.22	0.05	-0.33	-0.11	-0.16**
	$d_{2,3}$	Loneliness - Emotional competence	-0.08	0.09	-0.25	0.10	-0.03
	b_1	Stress – IAT	0.56	0.08	0.40	0.72	0.25**
	b_2	Loneliness - IAT	0.82	0.14	0.55	1.09	0.21**
	b_3	Emotional competence - IAT	-0.20	0.06	-0.31	-0.09	-0.12**
Total effect		c	5.27	0.58	4.14	6.41	0.32**
Direct efekt		c'	5.39	0.55	4.30	6.47	0.32**
Ind1	(a_1b_1)	Social media use – Stress –IAT	0.50	0.20	0.16	0.95	0.03*
Ind2	(a_2b_2)	Social media use – Loneliness - IAT	−0.20	0.12	−0.45	0.01	−0.01
Ind3	(a_3b_3)	Social media use – Emotional competence - IAT	−0.67	0.23	−1.14	−0.22	−0.04*
Ind4	($a_1d_{1,2}b_2$)	Social media use – Stress – Loneliness - IAT	0.22	0.09	0.07	0.41	0.01*
Ind5	($a_1d_{1,3}b_3$)	Social media use – Stress – Emotional competence - IAT	0.04	0.02	0.01	0.09	0.00*
Ind6	($a_2d_{2,3}b_3$)	Social media use – Loneliness - Emotional competence - IAT	0.00	0.01	−0.02	0.01	0.00
Ind7	($a_1d_{1,2}d_{2,3}b_3$)	Social media use – Stress – Loneliness - Emotional competence - IAT	0.00	0.01	−0.01	0.02	0.00

Model	Stress	Loneliness	Emotional competence	IAT
R^2	0.01	0.26	0.13	0.29
F	11.12**	128.25**	36.69**	74.94**
df	(1, 748)	(2, 747)	(3, 746)	(4, 745)

B = unstandardized regression coefficient SE = standard error; t = t value; 95% CILB = lower bound of the 95% confidence interval; 95% CIUB = upper bound of the 95% confidence interval; β = standardized regression coefficient; significance level; *p < .05, **p < .01; R^2 = coefficient of determination; F = F value; df = degrees of freedom of the model and the residual ($df_{1,2}$); IAT= Internet Addiction Test; Total Indirect effect: B = -0.11, SE = 0.40, 95% CI [-0.90; 0.70], β = -0.01

Table S3. Total, direct and indirect effects of online shopping or selling on problematic internet use via stress, loneliness, and emotional competence

			B	SE	LLCI	ULCI	β
	a_1	Online shopping or selling – Stress	1.58	0.27	1.05	2.11	0.21**
	a_2	Online shopping or selling – Loneliness	-0.27	0.15	-0.55	0.02	-0.06
	a_3	Online shopping or selling –Emotional competence	1.30	0.38	0.55	2.05	0.13**
	$d_{1,2}$	Stress – Loneliness	0.31	0.02	0.27	0.34	0.51**
	$d_{1,3}$	Stress – Emotional competence	-0.19	0.06	-0.30	-0.08	-0.14**
	$d_{2,3}$	Loneliness - Emotional competence	-0.11	0.10	-0.30	0.08	-0.05
	b_1	Stress – IAT	0.50	0.08	0.34	0.66	0.22**
	b_2	Loneliness - IAT	0.84	0.14	0.58	1.11	0.22**
	b_3	Emotional competence - IAT	-0.09	0.05	-0.20	0.01	-0.06
Total effect		c	6.80	0.58	5.66	7.95	0.39**
Direct efekt		c'	5.92	0.55	4.85	7.00	0.34**
Ind1	(a_1b_1)	Online shopping or selling – Stress –IAT	0.79	0.25	0.37	1.35	0.05*
Ind2	(a_2b_2)	Online shopping or selling – Loneliness - IAT	−0.23	0.13	−0.48	0.04	−0.01
Ind3	(a_3b_3)	Online shopping or selling – Emotional competence - IAT	−0.12	0.07	−0.27	0.03	−0.01
Ind4	($a_1d_{1,2}b_2$)	Online shopping or selling – Stress – Loneliness - IAT	0.41	0.11	0.21	0.65	0.02*
Ind5	($a_1d_{1,3}b_3$)	Online shopping or selling – Stress – Emotional competence - IAT	0.03	0.02	−0.01	0.07	0.00
Ind6	($a_2d_{2,3}b_3$)	Online shopping or selling – Loneliness - Emotional competence - IAT	0.00	0.00	−0.02	0.00	0.00
Ind7	($a_1d_{1,2}d_{2,3}b_3$)	Online shopping or selling – Stress – Loneliness - Emotional competence - IAT	0.00	0.01	0.00	0.02	0.00

Model	Stress	Loneliness	Emotional competence	IAT
R^2	0.04	0.26	0.04	0.31
F	34.03**	128.45**	9.34**	82.12**
df	(1, 748)	(2, 747)	(3, 746)	(4, 745)

B = unstandardized regression coefficient SE = standard error; t = t value; 95% CILB = lower bound of the 95% confidence interval; 95% CIUB = upper bound of the 95% confidence interval; β = standardized regression coefficient; significance level; *p < .05, **p < .01; R^2 = coefficient of determination; F = F value; df = degrees of freedom of the model and the residual ($df_{1,2}$); IAT= Internet Addiction Test; Total Indirect effect: B = 0.88, SE = 0.35, 95% CI [0.24; 1.61], β = 0.05
